# Restricted upper airway dimensions in patients with dentofacial deformity from juvenile idiopathic arthritis

**DOI:** 10.1186/s12969-022-00691-w

**Published:** 2022-04-27

**Authors:** Xiaowen Niu, Julianne Moland, Thomas Klit Pedersen, Anders Ellern Bilgrau, Paolo M. Cattaneo, Mia Glerup, Peter Stoustrup

**Affiliations:** 1 Section of Orthodontics, Aarhus University, Aarhus, Denmark; 2 Department of Oral and Maxillofacial Surgery, Aarhus University Hospital, Aarhus, Denmark; 3 Department of Mathematical Sciences, Aalborg University, Aalborg, Denmark; 4 Melbourne Dental School, Faculty of Medicine, Dentistry and Health Sciences, University of Melbourne, Australia, Formerly, Section of Orthodontics, Aarhus University, Denmark, Aarhus, Denmark; 5 Department of Pediatrics and Adolescent Medicine, Aarhus University Hospital, Aarhus, Denmark

## Abstract

**Background:**

This retrospective, cross-sectional study aimed to assess the pharyngeal airway dimensions of patients with juvenile idiopathic arthritis (JIA) and moderate/severe JIA-related dentofacial deformity (mandibular retrognathia/micrognathia), and compare the results with JIA patients with a normal mandibular appearance and a group of non-JIA patients.

**Methods:**

Seventy-eight patients were retrospectively included in a 1:1:1 manner as specified below. All patients had previously been treated at the Section of Orthodontics, Aarhus University, Denmark. All had a pretreatment cone beam computed tomography (CBCT). Group 1 (JIA+); 26 JIA patients with severe arthritis-related dentofacial deformity and mandibular retrognathia/micrognathia. Group 2 (JIA-); 26 JIA patients with normal mandibular morphology/position. Group 3 (Controls); 26 non-JIA subjects. Dentofacial morphology and upper airway dimensions, excluding the nasal cavity, were assessed in a validated three-dimensional (3D) fashion. Assessment of dentofacial deformity comprised six morphometric measures. Assessment of airway dimensions comprised nine measures.

**Results:**

Five morphometric measures of dentofacial deformity were significantly deviating in the JIA+ group compared with the JIA- and control groups: Posterior mandibular height, anterior facial height, mandibular inclination, mandibular occlusal inclination, and mandibular sagittal position. Five of the airway measurements showed significant inter-group differences: JIA+ had a significantly smaller nasopharyngeal airway dimension (ad2-PNS), a smaller velopharyngeal volume, a smaller minimal cross-sectional area and a smaller minimal hydraulic diameter than JIA- and controls. No significant differences in upper airway dimensions were seen between JIA- and controls.

**Conclusion:**

JIA patients with severe arthritis-related dentofacial deformity and mandibular micrognathia had significantly restricted upper airway dimensions compared with JIA patients without dentofacial deformity and controls. The restrictions of upper airway dimension seen in the JIA+ group herein were previously associated with sleep-disordered breathing in the non-JIA background population. Further studies are needed to elucidate the role of dentofacial deformity and restricted airways in the development of sleep-disordered breathing in JIA.

## Background

The temporomandibular joint (TMJ) is frequently involved in juvenile idiopathic arthritis (JIA) [1–4]. TMJ arthritis may lead to orofacial symptoms and dysfunction affecting health-related quality of life [1, 5–7]. TMJ involvement in skeletally immature patients may also impact dentofacial growth and development [8, 9]. The severity of JIA-related dentofacial growth disturbance depends on onset of TMJ involvement in relation to the mandibular growth trajectory [10, 11]. JIA-related dentofacial deformities span a continuum from minor dentofacial asymmetry to mandibular underdevelopment with a retrognathic position of the mandible referred to as “micrognathia” in the most severe form [8–10, 12].

The past decade has seen growing attention to the relationship between upper airway pharyngeal structures and dentofacial morphology; two-dimensional (2D) and recent studies three-dimensional (3D) [13–17] in the non-JIA background population have elucidated this relationship. Specific dentofacial morphological traits have been associated with reduced upper airway dimensions and resulting sleep-disordered breathing (SDB) in the background pediatric population. The notion is that the dentofacial skeleton serves as a scaffold for upper airway soft-tissue structures. The upper airways perform several physiologic functions including vocalization, swallowing, and respiration [18]. It stretches from the tip of the nose to the tip of the epiglottis or larynx, depending on the reference [19, 20]. In patients with mandibular retrognathia/micrognathia and a vertical mandibular growth pattern (e.g., steep occlusal plane), the retrusive mandibular position leads to a decreased intra-luminal diameter and increased upper airway resistance which, in turn, increases the risk of upper airway collapse, obstruction, and SDB [21–24]. SDB ranges from primary snoring at one extreme to complete upper airway obstruction at the other [25]. Pediatric sleep disturbances in the background population have a negative impact on children’s quality of life and physical and emotional well-being [26–29]. Sleep disturbances in school-aged children is a critical condition as good sleep hygiene is critical to behavior and intellectual performance [30]. Other recognized risk factors associated with childhood SDB in the background population are obesity, tonsillar, and adenoid hypertrophy [24].

The morphological traits associated with SDB in the background pediatric population are comparable to the dentofacial deformities in JIA patients with long-term TMJ involvement during growth. Further investigation of the relationship between JIA-related dentofacial morphology and upper airway dimensions therefore seems warranted since 1) JIA patients have a higher incidence of SDB and obstructive sleep apnea (OSA) than the background population without consistent indications of factors associated with sleep disorders in JIA [31–34], 2) TMJ involvement is a frequently occurring condition in JIA [1–4]. No research has yet elucidated the association between dentofacial deformity, upper airway dimensions, and SBD development in patients with JIA.

This study aimed to assess upper airway dimensions in patients with JIA-related dentofacial deformity and compare these patients with patients with JIA and normal facial morphology and healthy controls.

## Materials and method

### Population

This retrospective study included 78 patients divided into three groups: Group 1 (JIA+); JIA patients with arthritis-related mandibular retrognathia/micrognathia (*n* = 26). Group 2 (JIA-); JIA patients with normal mandibular appearance (*n* = 26) identified and matched to the JIA+ group by gender and age at a pretreatment radiological examination. Patients in the JIA+ and JIA- groups were previous or current patients affiliated with the Regional Craniofacial Clinic, Section of Orthodontics, Aarhus University, Denmark. We also included a gender- and age-matched control group (*n* = 26) of non-JIA subjects affiliated with the same institution that had received a cone-beam computerized tomography (CBCT) for orthodontic treatment planning of malocclusion.

Inclusion criteria for the JIA+ group: 1) Diagnosis of JIA according to the International League of Associations of Rheumatology (ILAR) criteria [35]; 2) presence of arthritis-related mandibular retrognathia/micrognathia based on findings from the clinical examination; 3) large-field-of-view CBCT taken in the 8–18-year age range before orthopedic/orthodontic or surgical treatment had been initiated; 4) no known previous tonsillectomy history.

Inclusion criteria for the JIA- group: 1) Diagnosis of JIA according to the ILAR criteria [35]; 2) no radiological signs of apparent arthritis-related dentofacial deformity based on findings from the clinical examination, 3) a large-field-of-view CBCT performed in the 8–18 year age range; 4) no known previous history of tonsillectomy.

Inclusion criteria for the Control group: 1) Non-JIA children and adolescents with a CBCT taken before orthodontic management with braces; 2) age 8–18 years; 3) no known previous history of tonsillectomy, primary snoring, or OSA; 4) no previous or current diagnosis of temporomandibular dysfunction.

Exclusion criteria for all three groups were: 1) Presence of dentofacial growth disturbances from underlying syndromes, traumas, or congenital birth defects involving the craniofacial or oropharyngeal area, 2) inadequate CBCT quality (e.g., low-quality CBCTs without clearly visualized airways or with significant artifacts.

The use of retrospective data from the electronic files of the three groups was approved by the The Danish Health Authorities and the Danish Data Protection Agency prior to initiation of the study.

### 3D Image processing

3D assessment of JIA-related dentofacial deformity and upper airway dimensions was obtained on institutional CBCT machines following the manufacturer’s instructions and the radiological CBCT protocol approved by the Danish Health Authority. The CBCT examinations were conducted using NewTom 3G or 5G machines (CEFLA s.c., Italy) with a 18 × 16 cm field of view. The image acquisition parameters included an approximate scanning time of 18 s with an active radiation of 3.6 s with settings of 110 kV and 3–7 mA. All CBCT scans were constructed with a 0.3 mm isotropic voxel dimension. Estimated radiation dose was 190 microSv. Scans were taken with the patient in a supine position. CBCT data obtained from CBCT scanning were exported in the DICOM (digital imaging and communications in medicine) format and imported into a specialized software program (Mimics 21.0, Materialise, Leuven, Belgium).

### Assessment of dentofacial deformity

**Fig. 1:**
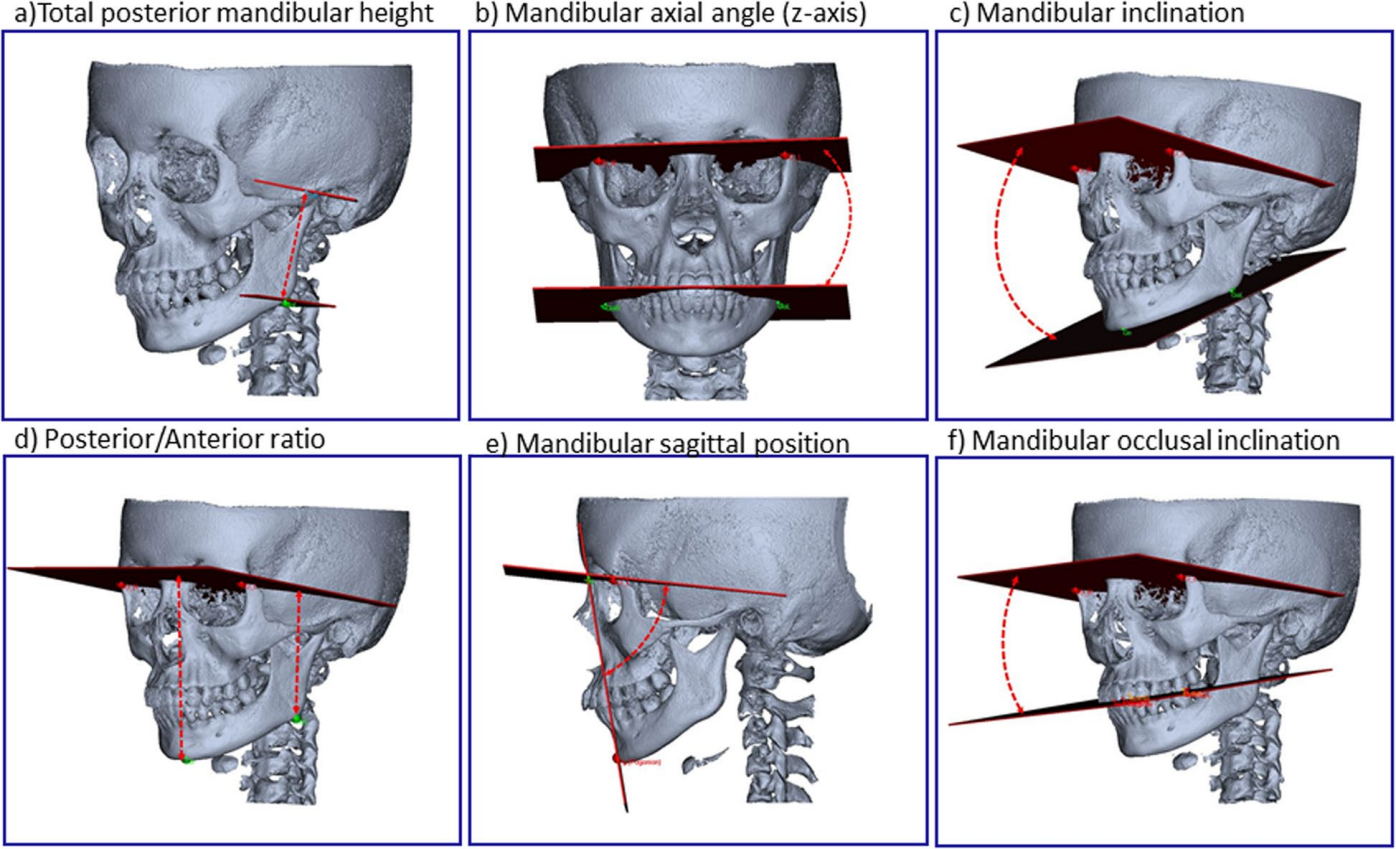
Morphometric measures used to assess dentofacial deformity

Assessment of dentofacial morphology and deformity was conducted following our previously published method[8] by which 3D information is obtained on mandibular sagittal position, vertical pattern, and asymmetries based on 23 anatomic landmarks, 12 internal planes, and six side-specific planes. For details about anatomical landmarks and constructed planes, we refer to Stoustrup et al. [8] and to the descriptions in the online supplemental material (S1, S2, S3, S4). The assessment of dentofacial deformities produces 21 morphometric outcome measures of dentofacial morphology of varying importance. In the present study, we included six of the original 21 outcome measures deemed of “high importance” for assessment of dentofacial deformity [8]. The included outcome variables were (Fig. 1a-f): total posterior mandibular height (inter-side difference to assess mandibular asymmetry), mandibular axial angle (facial asymmetry), mandibular inclination, posterior/anterior lower-face height ratio (anterior face height), mandibular sagittal position (degree of mandibular retrognathia), and mandibular occlusal inclination (steepening of the occlusal plane). Furthermore, we measured the maxillary inter-molar distance as an expression of maxillary width (transverse dimension).

### Assessment of upper-airway dimensions

In the present study, the term “upper airways” specifically refers to the “pharyngeal airways” not comprising the dimensions of the nasal cavity.

#### Upper airway dimensions – linear measurements and volumes

**Fig. 2:**
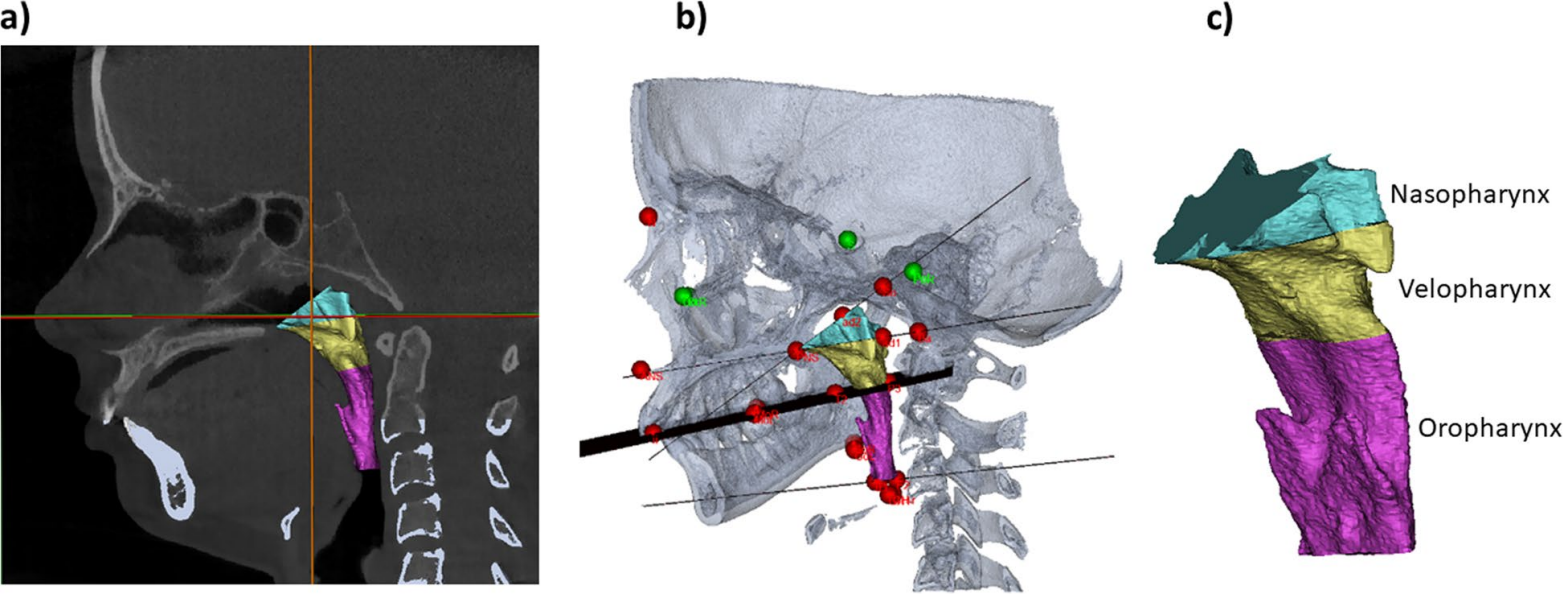
Upper airway dimensions. **a** and **b** Upper airways in relation to other anatomical structures. Green and red dots indicate anatomaical landmarks used for evaluation of dentofacial deformity. The black structure indicates the occlusal plane. **c** Subdivision of upper airways

To characterize the upper airway, 3D analysis was conducted using a slightly modified version of the method described by Di Carlo et al. 2015 [20]. The threshold levels used to generate the 3D reconstructions were determined individually for each CBCT dataset. The aim was to segment the upper airway to extract information about upper airway dimensions based on acknowledged linear measurements, total upper airway volume, and the partial volumes (i.e. lower nasopharynx, velopharynx, and oropharynx). Please see online supplemental material (S1, S2, S3, S4) for details about anatomical landmarks and outcome variables for upper airway assessment. The linear measurements outcome variables were: 1) upper sagittal dimension of the nasopharyngeal airway (termed “ad2-PNS” based on the anatomical landmarks involved), 2) lower sagittal depth of the nasopharyngeal airway (termed “ad1-PNS”). The airway volume outcome variables were: 3) total upper airway volume (mm^3^), 4) total surface area (mm^2^), 5) nasopharyngeal volume (mm^3^), 6) velopharyngeal volume (mm^3^), and 7) oropharyngeal volume (mm^3^) (Fig. 2).

#### Upper airway dimensions – Cross-sectional area and hydraulic diameter

To further elucidate the airway dynamics and the risk of upper airway resistance and risk of obstruction/collapse, we used two additional upper airway outcome variables: 8) The minimal cross-sectional area of the upper airway (CS) and 9) the minimal hydraulic diameter (D_H_) [36]. Both CS and D_H_ and are indicators of the upper airway intra-luminal space. Fluid dynamics and resistance to flow vary with the shape of a “pipe”. The shape of the upper airways is mostly irregular and not round. This change in shape greatly influences the CS, whereas the D_H_ may, to a greater extent, take into account the change in shape throughout the upper airways, which is an argument for including both measures [36]. Importantly, the positions of the minimal D_H_ and the minimal CS do not necessarily coincide in the same upper airway positions.

**Fig. 3:**
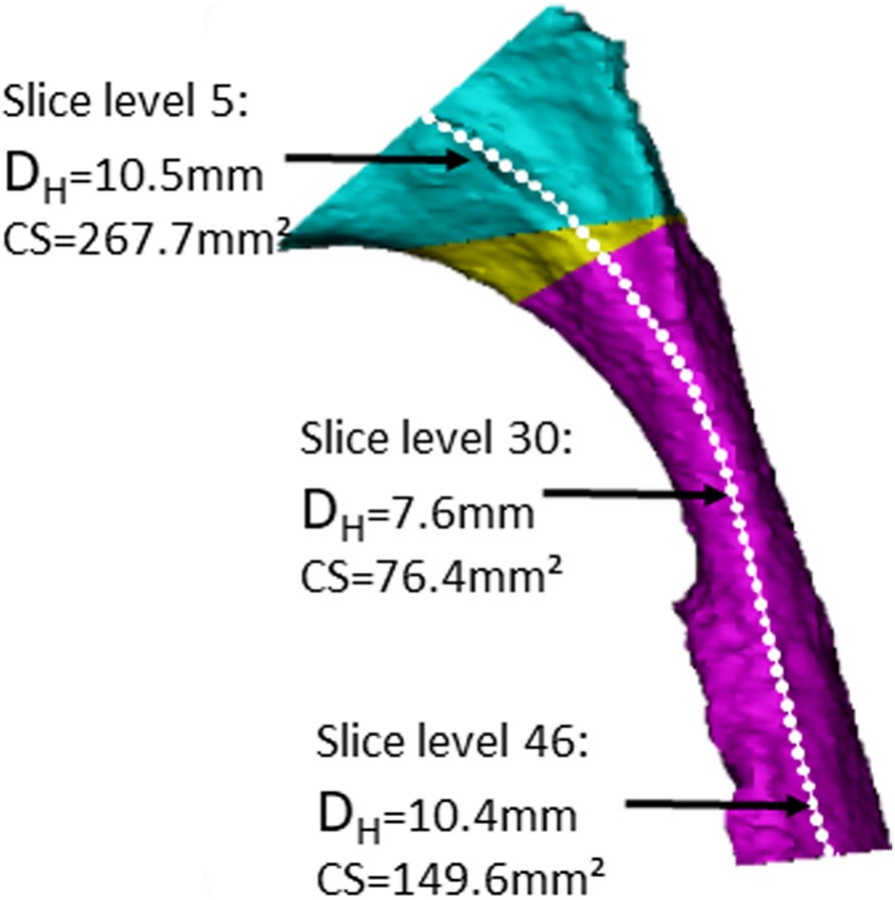
Assessment of cross-sectional area (CS) and hydraulic diameter (D_H_) along the centerline throughout the upper airway in a patient from the JIA+ group. Each white dot represents a slice level. The middle observation represents the position of the minimum CS and D_H_ (slice level 30). Notice how the upper assessment (slice level 5) and the lower assessment (slice level 46) have comparable D_H_ but great difference in CS due to difference in airway shape

The CS and D_H_ were assessed using the method described by Niu et al. [36] where an upper airway centerline is defined and consecutive “slices” are established perpendicular to the centerline to assess the CS and D_H_ at continuous positions (slice levels) along the upper airways. The CS and D_H_ were assessed on 50 consecutive slice levels from the top of the nasopharynx (slice 1) to the bottom of the oropharynx (slice 50). The CS and D_H_ were defined as the smallest value obtained throughout the course of the 50 slices on the centerline for each of the two variables (Fig. 3).

Duplicate assessments of outcome measures of dentofacial deformity and airway dimensions were conducted in a blinded fashion at a minimum three-week interval to assess intra-rater agreement. All measurements were made by the same operators (JM and XN).

### Statistics

An intra-class correlation coefficient (random effect model) was used to assess intra-rater reproducibility of the six variables representing dentofacial deformity and airway dimensions based on duplicate assessment of 24 random patients. Inter-group differences within each of the six outcome variables describing dentofacial deformity and the seven variables describing airway dimensions (excluding CS and D_H_) were assessed using the Kruskal Wallis test as the primary test. Outcome variables with a significant inter-group difference in the primary Kruskal Wallis test were further analyzed with the Wilcoxon signed-rank post-test. The level of significance was set at *p* = 0.05.

#### Statistical assessment of cross-sectional area and minimal hydraulic diameter

The interaction of the 50 consecutive slices of cross-sectional areas and hydraulic diameters throughout the upper airways calls for a more complex statistical evaluation. Plots and descriptive statistics (mean and standard error (SE)) were used to summarize the data across slices for each measure and group. Generalized additive models (GAM) smoothing estimates of mean curves were created to investigate mean curves for each measure and group. Differences between groups were estimated using three different classes of regression models of varying complexity: 1) multiple linear regression, 2) non-linear (spline) regression, and 3) mixed effect models [37, 38].

*Multiple linear model:* We first employed the simplest linear model suitable for the data. Each response measure was analyzed independently using a linear regression with group, slice, and their interaction as explanatory variables allowing for an ANOVA test of a group effect on the measure. This model corresponds to straight mean curves (lines) of varying slope and intercept in each group. This model thus ignores any non-linearity in the mean curves and subject-specific effects; therefore, we included another regression model.

*Non-linear model:* To improve the data model and account for non-linearity in the mean curves, we also fitted a b-spline regression model with equidistantly placed knots along the slices and the group variable as an explanatory variable. Graphically, this models the mean curves as non-linear slice curves/functions. However, it allows only translation (up or down) of the mean- curve across groups. This restriction only enables tests for a difference in mean curve values across groups.

*Mixed effect model:* The former two regression models do not take subject-specific effects into account. To adjust for this effect more appropriately, we fitted a mixed effect model with a mixed effect term corresponding to each subject. This model corresponds to each subject curve as being translated up or down in relation to the patients’ group mean (i.e. a random-intercept model in each group).

*Non-linear mixed effect model:* We combined the non-linear and mixed effect model and included other covariates (sex and pre-treatment age) to fully model all aspects of the dataset.

## Results

Cohort characteristics are presented in Table 1. The three groups were equally distributed in terms of gender and age (Table 1). Intra-class correlations for duplicate measurements are presented in Table S5. All variables tested had an acceptable intra-rater reproducibility.

**Table 1:** Cohort characteristics

	**JIA with dentofacial deformity (JIA+)**	**JIA without dentofacial deformity (JIA-)**	**Controls**
Number	26	26	26
Females (percentile)	20 (76.9%)	20 (76.9%)	20 (76.9%)
Mean age at baseline, years ± SD	11.63 ± 1.67	11.58 ± 1.59	11.56 ± 1.07
**JIA subcategories:**			
Oligoarticular	15 (57.7%)	13 (50%)	-
Polyarticular	7 (26.9%)	7 (26.9%)	-
Systemic	1 (3.9%)	1 (3.9%)	-
Psoriatic	1 (3.9%)	1 (3.9%)	-
Enthesitis-related arthritis	1 (3.9%)	3 (11.5%)	-
Unspecified	1 (3.9%)	1 (3.9%)	-

### Dentofacial deformity

**Fig. 4:**
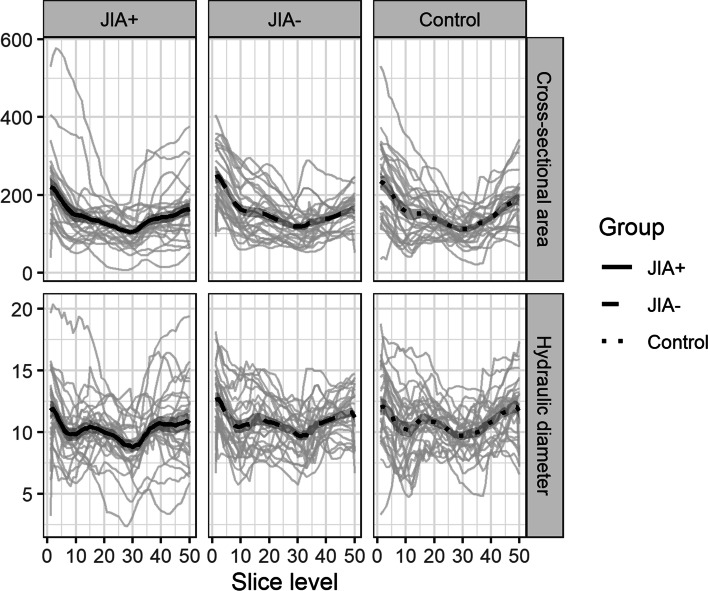
Average cross-sectional areas (mm^2^) and hydraulic diameter (mm) throughout the upper airways from each of the groups. Group 1 (JIA+), group 2 (JIA-), group 3 (controls). Solid black lines represent the mean curve values. The grey lines indicate intra-group curve values for each subject

**Fig. 5:**
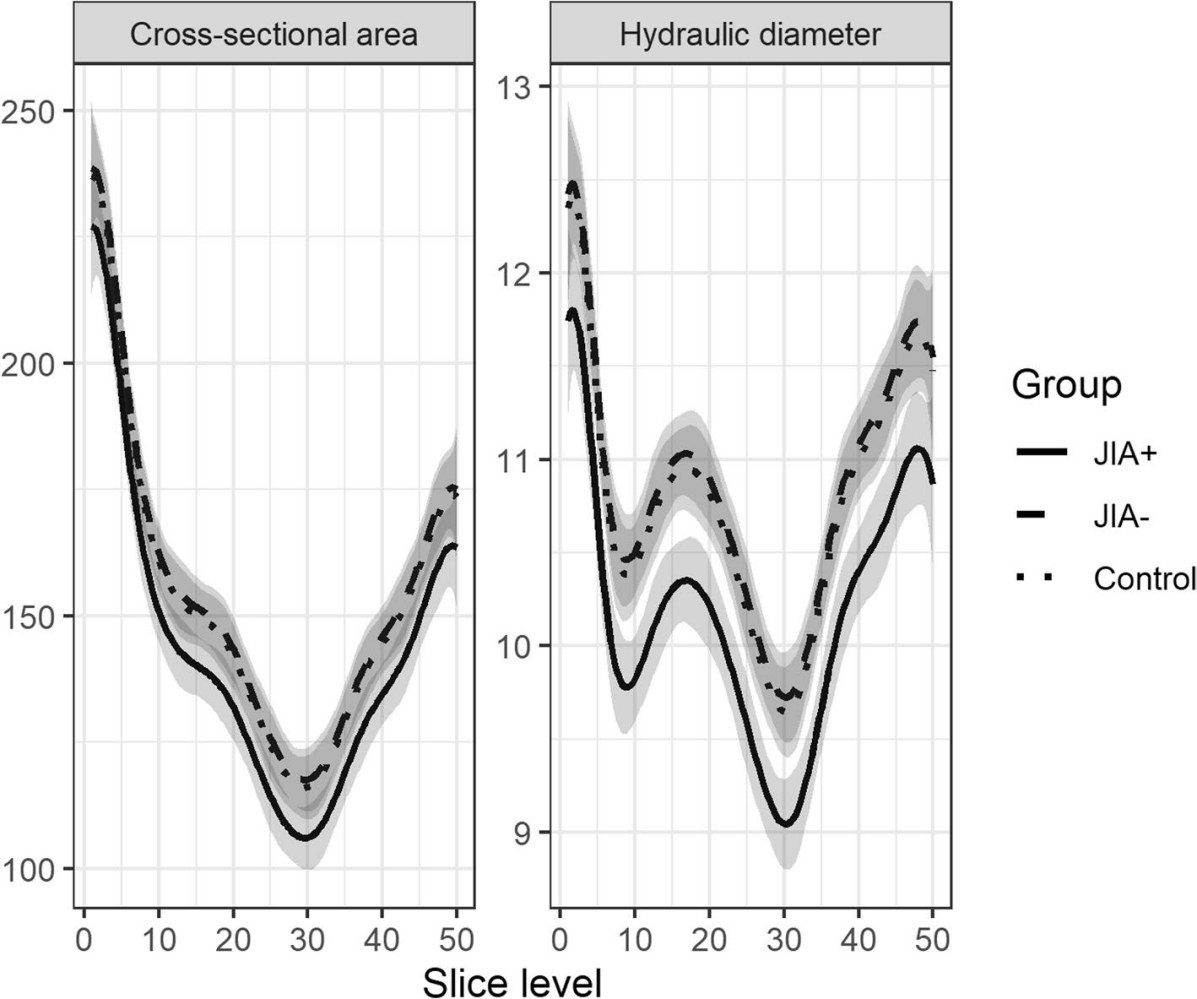
Comparison of cross-sectional areas (mm^2^) and hydraulic diameter (mm) throughout the upper airways from each of the groups. Group 1 (JIA+), group 2 (JIA-), group 3 (controls)

Primary testing showed significant inter-group differences in five of the six morphometric dentofacial deformity outcomes (Table 2); total posterior mandibular height, mandibular inclination, posterior/anterior face height, mandibular sagittal position, and mandibular occlusal inclination (Fig. 1). For the five variables with a significant inter-group difference in the primary test, a secondary post-test (Wilcoxon signed-rank test) revealed that the JIA+ group was characterized by: 1) a significantly larger degree of facial asymmetry due to inter-side posterior mandibular height differences (Fig. 1a); 2) reduced mandibular vertical growth and development illustrated by a significantly larger anterior face height (Fig. 1d); 3) significantly larger mandibular inclination values (Fig. 1c); 4) mandibular occlusal inclination (Fig. 1f); 5) mandibular retrognathia illustrated by a significantly reduced mandibular sagittal position (Fig. 1e) in the JIA+ group. No significant difference for any measurement was found between JIA- and controls.

**Table 2:** Inter-group comparison of dentofacial deformity and dimension of airways. *Negative values indicate degree of inter-side asymmetry in millimeters. **Post-tests were conducted only for variables with a significant inter-group difference in the primary test

	**Group 1 (JIA+):** **JIA with dentofacial deformity** **Median (25–75 quartile)**	**Group 2 (JIA-): JIA without dentofacial deformity** **Median (25–75 quartile)**	**Group 3 (Ctr):** **Controls** **Median (25–75 quartile)**	**Primary statistical test**	Post-tests**
**Skeletal and dental variables**					
Total posterior mandibular height*	-2.18 (-5.42/-1.18)	-1.06 (-1.69/-0.65)	-1.06 (-2.22/-0.47)	0.01	JIA+ < JIA- = Ctr
Mandibular axial angle	1.4 (0.7/2.88)	1 (0.63/1.48)	0.81 (0.52/1.66)	0.14	
Mandibular inclincation	42.13 (30.5/47.86)	30.88 (28.12/32.78)	27.8 (25.52/32.32)	0.0001	JIA+ > JIA- = Ctr
Posterior/anterior face height	0.66 (0.62/0.7)	0.71 ( 0.69/0.73)	0.73 (0.7/0.76)	0.0001	JIA+ < JIA- = Ctr
Mandibular sagittal position	72.1 (67.65/78.53)	79.8 (77.93/81.58)	81.1 (78.98/84.93)	0.0001	JIA+ < JIA- = Ctr
Mandibular occlusal inclination	22.79 (14.28/26.86)	14.72 (12.87/17.06)	12.2 (7.91/16.06)	0.0001	JIA+ > JIA- = Ctr
Inter-molar distance	47.12 (46.21/47.86)	47.945 (45.68/49.17)	47.03 (45.08/48.88)	0.46	
**Airways**					
Total airway volume (mm^3^)	6563.06 (5942.79/8091.28)	6880.62 (5511.17/8211.9)	6983.79 (5702.67/9991.34)	0.80	
Total surface area (mm^2^)	3205.54 (3015.53/3803.66)	3322.72 (2902.79/3571.65)	3529.83 (2992.73/4255.86)	0.55	
Nasopharynx volume (mm^3^)	1271.71 (947.78/1640.03)	1352.62 (1087.83/2242.37)	1306.63 (659.22/2182.89)	0.71	
Velopharynx volume (mm^3^)	1303.48 (746.72/2116.85)	2224.82 (1531.72/2777.82)	2111.2 (1549.33/3247.99)	0.006	JIA+ < JIA- = Ctr
Oropharynx volume (mm^3^)	4189.35 (3348.96/5087.88)	2935.03 (2464.15/3531.92)	3719.31 (2881.26/4477)	0.003	JIA+ > JIA- = Ctr
Adn1-Pns (mm)	19.19 (17.97/22.98)	21.36 (17.46/24.59)	21.74 (17.84/25.49)	0.38	
Adn2_Pns (mm)	13.42 (11.98/15.56)	15.08 (13.29/19)	16.95 (13.09/18.7)	0.03	JIA+ < JIA- = Ctr

### Upper airway dimensions – linear measurements and volumes

Primary testing showed significant inter-group differences for upper airway dimensions (Table 2): 1) upper sagittal depth of the pharyngeal airway (ad2-PNS); 2) partial velophargyngeal volumes; 3) partial oropharyngeal volumes. Furthermore, post-testing illustrated that JIA+ had a significantly lower pharyngeal airway (Ad2-PNS distance) depth than JIA- and controls. The velopharygeal volume was significantly smaller in JIA+ than in JIA- and control subjects. The oropharyngeal volume was significantly larger in the JIA+ than in JIA- and control subjects. No significant difference in upper airway dimensions was found between JIA- and control subjects.

### Upper airway dimensions – minimal cross-sectional area and hydraulic diameter

*Descriptive statistics*: Fig. 4 shows the mean curves and the intra-group variation of the consecutive measures of hydraulic diameters and the cross-sectional area in each group. Most notably, the mean curves appear similar in shape throughout the course of the upper airways despite considerable inter-subject variation. On Figs. 4 and 5, the JIA+ group values for CS and D_H_ appear consistently lower than the values for the JIA- and controls for both measures. The minimal CS and D_H_ were positioned at similar locations on the curves (slice level 29 to 31 on the centerline) (Fig. 5), which is a location in the oropharynx/velopharynx. The correlation between the two outcome measures CS and D_H_ reveal a high correlation of 0.89 across groups with similar within-group correlations.

*Linear model:* On average, the model estimated an average 28.04 mm^2^ difference for CS and 0.83 mm difference between JIA+ and JIA- for D_H_. The corresponding mean difference between controls and JIA+ groups was 10.61 mm^2^ for CS and 0.44 mm for D_H_. The ANOVA hypothesis test of no differences in mean values across groups was significant for both CS (*p* = $1.74\times {10}^{-5}$) and D_H_ (*p* = $9.36\times {10}^{-14}$) indicating a significant smaller airway dimensions in JIA+ compared to the other two groups.

*Non-linear model*: The non-linear model estimated a mean inter-group difference from JIA- to JIA+ of 11.51 mm^2^ for CS and 0.68 mm for D_H_. The corresponding mean inter-group difference between JIA+ and controls was 9.98 mm^2^ for CS and 0.6 mm for D_H_. The results from the non-linear spline regressions model showed significant inter-group results (*p* = $2.47\times {10}^{-6}$ for CS and *p* = $8.28\times {10}^{-15}$ for D_H_) when the restrictive assumption of linear mean curves was relaxed. This supports that upper airway dimensions in JIA+ was significantly reduced compared to the other two groups.

*Mixed effect model*: The mixed effects model estimated a mean difference from group JIA+ and JIA- of 28.04 mm^2^ for CS and 0.83 mm for the D_H_. The corresponding mean differences between controls and JIA was 10.61 mm^2^ for CS and 0.44 mm for D_H_. In the mixed effects model, the overall estimations were comparable to earlier results but did not remain significant (*p* = $0.126$ for CS, *p* = $0.257$ for D_H_) except for inter-group difference in minimal CS between JIA+ and JIA- (*p* = 0.048).

**Fig. 6:**
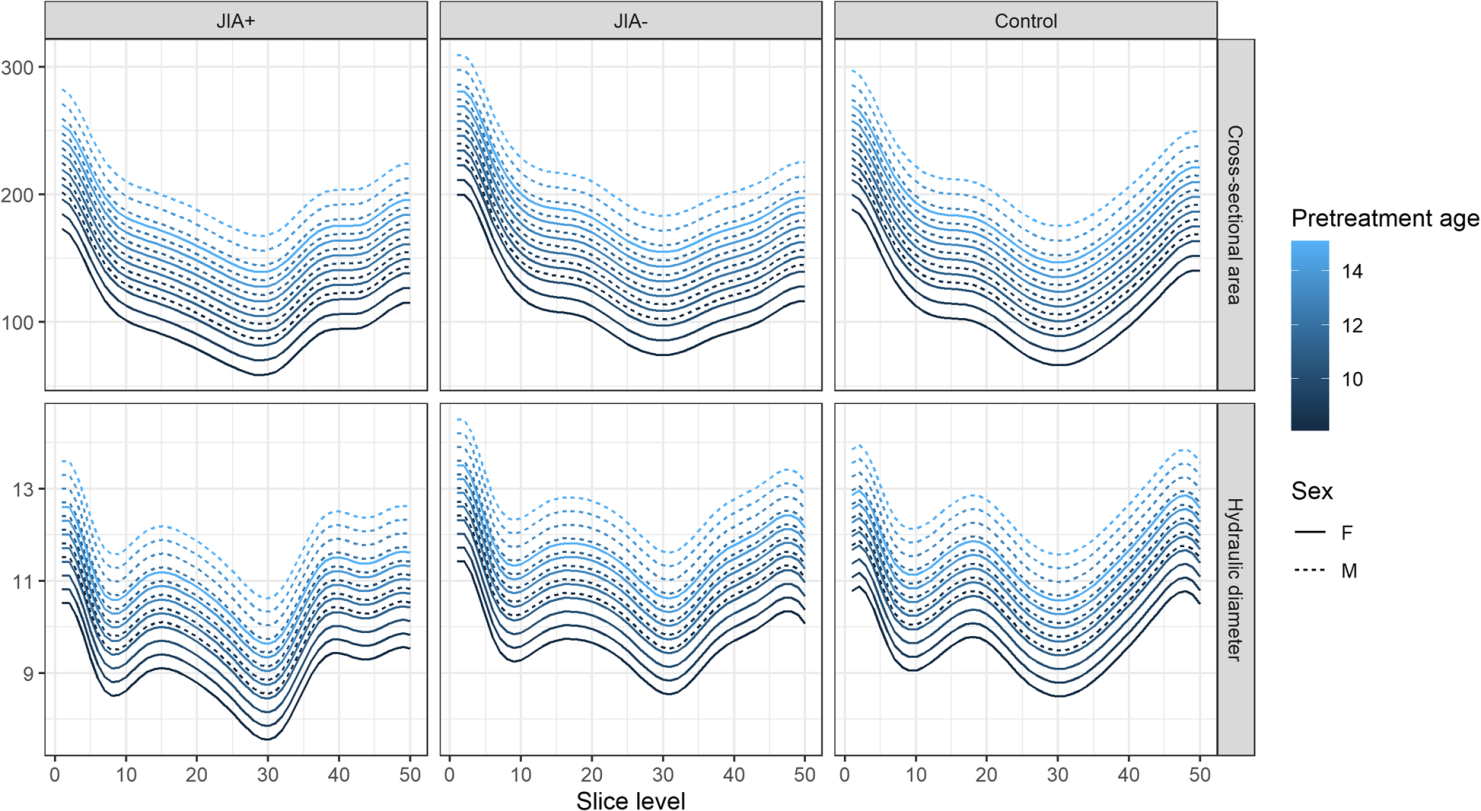
Non-linear mixed effect model combining covariates of sex and pre-treatment age with average cross-sectional area (mm^2^) and hydraulic diameter (mm) within the three groups. Group 1 (JIA+), group 2 (JIA-), group 3 (controls)

*Non-linear mixed effect model*: Mean values as fitted in the non-linear mixed effect model are shown in Fig. 6. While the inter-group comparisons remain largely as in the linear mixed effect model, the models estimates, for each additional year of age at pretreatment, displayed a change of 11.55 mm^2^ (*p* < 0.05) for minimal CS and 0.3 mm (*p* < 0.05) for D_H_. Likewise, the estimated difference from boys to girls was -28.31 mm2 for minimal CS (*p* < 0.05) and -0.99 mm for D_H_ (*p* < 0.05). As seen in Fig. 6, a 3 year older subject has on average the same effect on minimal CS and minimal D_H_ as the difference between boys and girls.

In summary, data for CS and D_H_ were found to be inconsistent with the null-hypothesis of no mean difference between the three groups in the various models applied. The shortcomings of the assumptions in the parsimonious linear model were examined through three, more advanced, models with consistent and largely unchanged results in terms of effect estimates. The JIA+ groups seems to have significant smaller upper airway dimensions when compared to JIA- and controls. However, significant group-differences did not remain significant across all estimates when accounting for repeated measure within each subject in the mixed effect model, though the data seems to show an inter-group difference (Fig. 5). No indications of significant inter-group differences between JIA- and controls were found.

## Discussion

To our knowledge, this is the first study to investigate the relationship between dentofacial deformity and upper airway dimensions in JIA subjects. The principal findings of this study were: 1) Upper airway dimensions and morphology vary greatly between individuals and within the three defined groups. 2) Subjects with JIA and related dentofacial deformity (e.g. reduced mandibular dimensions and steep mandibular/occlusal planes) display significantly restricted upper airway dimensions compared with JIA subjects with average facial morphology (JIA-) and controls. 3) The average cross-sectional areas and hydraulic diameters are restricted in most of the length of the upper airways in subjects with JIA-related dentofacial deformity compared with the two other groups. However, the average position of the minimal CS and D_H_ were found in the same position within the oropharyngeal area in all three groups. 4) Subjects with JIA and no signs of dentofacial deformity have upper airway dimensions comparable to those of healthy controls. We believe that our findings are of considerable clinical interest, since subjects with JIA have a higher risk of SDB than the background population. However, the complex relationships between JIA and SDB remain unsolved [31, 32, 34].

### Dentofacial deformity and upper-airway dimensions

The morphological signs of dentofacial deformity seen in the JIA+ group herein are comparable with previous findings [8–12]. We believe that the severity of the deformity in the JIA+ group may be characterized as “moderate/severe” on a continuum of JIA-associated dentofacial deformity.

Controversy prevails as to the relationship between facial morphology and upper airway dimensions. In our study, the upper airway dimensions were restricted in the JIA+ group and inter-subject variations were large in all three groups (Fig. 4). This corroborates previous studies investigating this relationship in non-JIA subjects [15, 16, 39–41]. Kim et al. [41] found a significantly smaller total upper airway volume in preadolescent subjects with retrognathic mandibles than in subjects with a normal posterior-anterior skeletal relationship. El and Palomo [16] found that subjects with mandibular retrognathia had significantly smaller oropharyngeal airway volumes than subjects with an average mandibular position (Class I) and subjects with protrusive mandibular position (Class III).

### Restricted upper-airways and the risk of sleep-disordered breathing

The findings of the present study may indicate that the JIA+ group is at risk of developing SDB. The dimension of the pharyngeal airway (Ad2-PNS distance) was reduced in the JIA+ group. A reduced Ad2-PNS distance has been related to pediatric OSA in non-JIA population in a systematic review by Katyal et al. [22]. In addition, the velopharyngeal volume was reduced in the JIA+ group compared with the other two groups. Conversely, the oropharyngeal volume was increased in the JIA+ group compared with the other groups. This is an inconsistent finding probably related to the technical subdivision of the upper respiratory tract and the difference in length/morphology in the JIA+ group.

Other indications of a SDB risk are that the JIA+ group had an increased lower-face height, a retrognathic mandibular position, and increased mandibular and occlusal inclination. According to a systematic review and meta-analysis [42], these morphological traits are risk factors for development of pediatric OSA. Future research is warranted to elucidate the role of dentofacial morphology in subjects with JIA and SDB.

### Implication of results

We used the 3D CBCT technique to capture the dimensions of the upper airway structures and facial morphology. The CBCT technique is considered a reliable method to assess upper airway dimensions [43, 44]. The importance of the third dimension was previously emphasized by Lenza et al. who stated that adequate the upper airway assessment requires the combination of linear measurements, area, and volume as no single volume or linear measurement alone depicts the actual airway morphology [45]. To capture the airway complexity, we assessed the CS and D_H_ along the predefined centerline throughout the upper airways. This illustrated that the CS and D_H_ vary along the centerline in the same form (shape of mean curves) with the JIA+ group displaying lower mean values throughout most of the upper airways (Fig. 5) than the other groups. Future research should study the implication of these results. Our results are notable because they coincide with findings by Arens et al. who examined the cross-sectional area of the upper airways using magnetic resonance imaging (MRI) in children with OSA and healthy controls [46]. Arens et al. found that the upper airway cross sectional area varies along the centerline between children with OSA and healthy controls with significant airway narrowing occurring continuously throughout most of the length of the upper airways in the OSA group. Arens et al. speculated that the continuous narrowing of the upper airways may be essential to the air flow resistance that characterizes subjects with OSA [33].

OSA is at the extreme end of the manifestation of SDB and has received attention in JIA research [32, 33]. OSA is characterized by increased upper airway resistance causing partial (hypopnea) or complete collapse (apnea) of the upper airways followed by increased respiratory efforts, arousals, and sleep fragmentation [47]. The findings of the present study fuel the hypothesis that JIA-associated dentofacial deformity may restrict the upper airways, which, in turn, may increase the risk of upper airway collapse. According to Susarla et al., the patency of the flexible structure of the upper airways is ensured by a balance between collapsing forces and dilating forces [23]. Collapsing forces include increased intra-luminal pressure from narrow airways and increased extra-luminal pressure from the surrounding pharyngeal related to soft tissue or certain skeletal traits [23]. According to Susarla et al., resistance to airflow is inversely proportional to the fourth power of the radius of the upper airway [23]. A decrease in upper airway cross-section therefore significantly affects airflow resistance. We therefore hypothesize that the reduced CS and D_H_ in the JIA+ group of the present study may predispose to development of OSA based on the following notion; JIA-associated dentofacial deformity leads to upper airway narrowing followed by increased intra-luminal resistance, which, in turn, may decrease airflow and act as collapsing force on the flexible structures of the upper airways. In connection with this hypothesis, reservations must be made that OSA consists of a complex interplay between anatomical conditions, the central nervous system, and sleep-related conditions [23, 47]. To characterize OSA as an “anatomical condition” alone seems inconsistent with contemporary views.

### Limitations and strengths

**Fig. 7:**
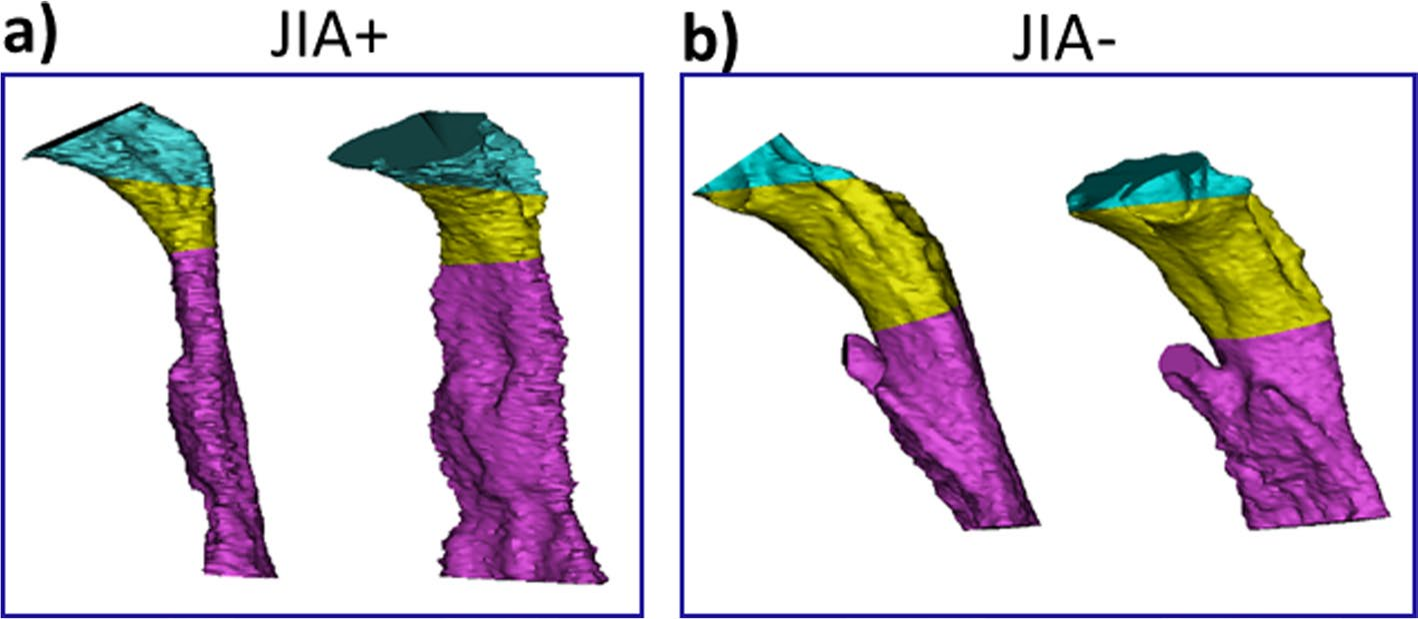
An illustration of inter-group differences in upper airway morphology between JIA+ and JIA-. Airways are displayed in lateral and oblique views. a) Upper airway in a patients from the JIA+ group (total volume 8,091.28 mm^3^). B) Upper airway in a subject from the JIA- group (total volume 8,096.69 mm^3^). Visual differences are appreciated in sagittal and vertical dimensions of the airways despite comparable total volumes; the JIA+ upper airway presents as a “long and narrow” upper airway

Our study has certain limitations that require further consideration: 1) Lowe et al. [48] reported changes in airway dimensions during the respiratory phases. However, due to the retrospective nature of our study, no control for respiratory movements (inspiration, resting, exhalation) was conducted during the CBCT acquisition. This is a limitation of the study as volume changes related to respiration phases may potentially present as systematic errors [48, 49]. 2) Another technical limitation relates to the radiological plane used for subdivision for the inferior border for the velopharynx and the superior border for the oropharynx. This plane was greatly affected by the inclination of the occlusal plane (Fig. 2b), giving the incorrect impression that the JIA+ group (with mandibular retrognathia) had a larger oropharyngeal volume than the JIA- group and controls due to a steep occlusal plan in the JIA+ group. Importantly, the volume does not inform about the airways’ regional shape, so that only by looking at the pharyngeal airway volumes would give misleading information about the airway patency and breathing ability of the subjects. 3) No group-differences remained significant across all estimates when accounting for repeated measure within each subject in the mixed effect model. We attribute the lack of inter-group significance from this model to a lack of power (small number of subjects and thus independent observations) and large inter-subject variation rather than a true lack of differences. We hypothesize that inclusion of a larger cohort would have generated a test result in line with the significant results obtained in the non-linear model and the mixed-effect model d. 4) No global agreement exists on the definition of the superior and inferior limits of the upper airways or the demarcation for partial volumes. This gives rise to different airway volumetric subdivisions among studies and hampers comparisons between studies, especially partial volume comparisons, and generation and comparison with normative values. In general, the use of volumes as an outcome measure must be considered a potential source of error as comparable volumes may be found in airways with considerable variation in shape (Fig. 7).

Important strengths of this study are: 1) the use of validated 3D assessment of the dentofacial morphology and pharyngeal airway dimensions, 2) the fact that the CBCTs were taken with the patient in a supine position, which mimics the sleeping position, and 3) the inclusion of a JIA group without dentofacial deformity and a non-JIA control group. The inclusion of three groups (JIA+ , JIA-, controls) made it possible to compare dentofacial and airway morphology between subjects with JIA and healthy controls, but also between subjects with JIA with/without dentofacial deformity which is a strength to our observations.

## Conclusion

In summary, JIA patients with moderate to severe dentofacial deformity have significantly restricted upper airway dimensions compared with JIA patients without dentofacial deformity and non-JIA controls. The restrictions of the upper airways seen in subjects with dentofacial deformity herein have previously been associated with SDB in the non-JIA background population. Further studies are needed to elucidate the role of dentofacial deformity and restricted upper airways in the development of sleep-disordered breathing in JIA.

## Data Availability

The data that support the findings of this study is considered third-party patient-owned data By Danish regulations. Restrictions apply to the availability of these data, which were used under license for the current study, and so are not publicly available. Data are however available from the authors upon reasonable request and with permission of Danish Health Authorities in the Central Denmark Region (www.stps.dk, case: 3–3013-2558/1).

## Abbreviations

ad1-PNS: Lower sagittal depth of the nasopharyngeal airway
ad2-PNS: Upper sagittal dimension of the nasopharyngeal airway
ANOVA: Analysis of variance
CBCT: Cone-beam computerized tomography
CS: Minimal cross-sectional area
D_H_: Minimal hydraulic diameter
DICOM: Digital imaging and communications in medicine
GAM: Generalized additive models
ILAR: International League of Associations for Rheumatology
JIA: Juvenile idiopathic arthritis
JIA+: Subjects with arthritis-related mandibular retrognathia/micrognathia
JIA-: Subjects with normal mandibular appearance
OSA: Obstructive sleep apnea
SE: Standard error
SDB: Sleep-disordered breathing
TMJ: The temporomandibular joint
2D: Two-dimensional
3D: Three-dimensional
